# Haematological Features of White Rats *(Rattus norvegicus)* Infected with *S. pyogenes* and Administered with Probiotics (Yogurt)

**DOI:** 10.1155/2022/2899462

**Published:** 2022-06-30

**Authors:** Novina Rahmawati, Maimun Syukri, Darmawi Darmawi, Indra Zachreini, Utari Zulfiani, Muhammad Yusuf, Rinaldi Idroes

**Affiliations:** ^1^Graduate School of Mathematics and Applied Sciences, Universitas Syiah Kuala, Banda Aceh 23111, Indonesia; ^2^Medical Faculty, Universitas Syiah Kuala, Banda Aceh 23111, Indonesia; ^3^Faculty of Veterinary Medicine, Universitas Syiah Kuala, Banda Aceh 23111, Indonesia; ^4^Medical Faculty, Universitas Malikussaleh, Aceh Utara, Indonesia; ^5^Department of Chemistry, Faculty of Mathematics and Natural Sciences, Universitas Syiah Kuala, Kopelma Darussalam, Banda Aceh 23111, Indonesia; ^6^Department of Pharmacy, Faculty of Mathematics and Natural Sciences, Universitas Syiah Kuala, Banda Aceh 23111, Indonesia

## Abstract

This study aimed to study the inhibition activity of lactic acid bacteria probiotics deriving from Acehnese fermented Etawa goat's milk (yogurt) against *Streptococcus pyogenes* bacterial infection in rats (*Rattus norvegicus*). Haematological analysis of the rats' blood was performed on the following parameters: platelets, leukocytes, lymphocytes, neutrophils, and monocytes, where the data were further processed using ANOVA and Duncan's test with a confidence level of 95% (0.05). The results revealed that administering yogurt containing probiotics could reduce infections in the throats of rats caused by *S. pyogenes*. Based on the haematology examination, the probiotic yogurt could maintain the number of platelets, leukocytes, lymphocytes, neutrophils, and monocytes. Statistical significance was obtained when the infected rats were administered with a ±1.00 mL/day dose for seven days of treatment (*p* < 0.05).

## 1. Introduction

The use of antibiotics to treat infectious diseases in humans could cause adverse reactions and induce the emergence of antibiotic-resistant pathogens [1]. One of the diseases commonly treated using antibiotics is a sore throat caused by irritation or inflammation in pharyngitis or tonsillitis [2]. It is due to the fact that the disease is a common manifestation of *Streptococcus pyogenes* bacterial infection [2]. To overcome the antibiotic resistance in streptococci, the administration of probiotics (i.e., *Lactobacillus* probiotics) could be used as an integrative therapy [3]. Lactic acid bacteria are microflora classified as probiotics which could be obtained from fermentation [4]. The isolated lactic acid bacteria have several advantages as a probiotic including their survivability, reproducibility, and secretion of antibacterial substances (which could inhibit enteric gut bacteria) [5]. *Lactobacillus* and *Bifidobacteria* are the most common lactic acid bacteria used as probiotic microorganisms [5].

Food and beverages containing probiotics have many health benefits, such as helping the digestive system process and absorption of nutrients [6]. Moreover, such probiotic products could inhibit the pathogen in the digestive tract (i.e., *Escherichia coli*, *Streptococcus aureus*, *Salmonella typhimurium*, *Vibrio cholerae*, and *Mycobacterium tuberculosis*) [6]. In addition, consuming food or beverages with probiotics could prevent constipation, cancer, excessive blood cholesterol level, lactose intolerance, and increase immune response [6]. Based on a previous report [7], ten local lactic acid bacteria species were extracted from raw beef showing probiotic properties by producing antimicrobial compounds. Another source to obtain lactic acid bacteria is yogurt, in which the yogurt made of Etawa goat's milk has been reported to contain Gram-positive isolates with a negative catalase test [8]. Further investigation under the microscope in the foregoing report revealed the shape of the bacillus cells suggesting the milk isolate contained *Lactobacillus* bacteria [8].

Nonetheless, the administration of probiotic bacteria via the oral route was reported to affect the body's metabolic system [9], including the haematological status [10]. Dysregulation of blood parameters could adversely affect blood function. Hence, it is important for researchers to investigate the effect of probiotic administration on haematological blood parameters including the number of platelets, leukocytes, lymphocytes, neutrophils, and monocytes.

Herein, the objective of this study is to investigate the ability of local probiotics (in the form of lactic acid bacteria) derived from fermented Etawa goat's milk (yogurt) procured from Kopelma Village, Banda Aceh, Aceh Province, Indonesia, to treat *S. pyogenes* infection-induced inflammation in the throat of the rat model. The changes of haematological profiles (platelets, leukocytes, lymphocytes, neutrophils, and monocytes) following the probiotic therapy were also investigated.

## 2. Materials and Methods

### 2.1. Experiment Animals and Feeding

Twenty male rats (*Rattus norvegicus*) aged 5-6 weeks with a bodyweight range of 115–135 grams were obtained from the Test Animal Laboratory, Faculty of Veterinary Medicine, Universitas Syiah Kuala. The rats were divided into four treatment groups comprising five rats/group (K0 (negative control) and K1-3). Before treatment, all rats were acclimated for three days and fed *ad libitum*. The treatment process is presented in Table 1.

### 2.2. *S. pyogenes* Bacterial Infection

The rats were prepared to be infected with *S. pyogenes* bacteria. The culture of *S. pyogenes* (ATCC 12344) used was collected from the Laboratory of Oral Biology, Faculty of Dentistry, Universitas Indonesia, Jakarta. *S. pyogenes* infection was carried out using a population of 0.5 McFarland bacteria (1.5 × 10^8^ CFU/mL) administered orally (swab) using a probe.

### 2.3. Yogurt Administration

Yogurt was orally administrated to the K2 group on the second day for the following 13 days, whilst the administration was carried out on the first day for the following 7 days for the K3 group. The administration was carried out by force-feeding 1 mL yogurt via injection to the mouth of the rat into the throat using a gastric probe.

### 2.4. Blood Sampling and Hematological Analysis

Blood samples were drawn from the rat through the lateral vein in the tail [12], and taken on days 1, 7, and 14. Each sample was inserted in a tube which had been priorly added with EDTA and subsequently analysed for *S. pyogenes* parameters [10]. Haematological observation was performed on the blood components including the number of platelets, leukocytes, lymphocytes, neutrophils, and monocytes.

### 2.5. Data Analysis

The data obtained in this study were analysed statistically using ANOVA (analysis of variance) after the Shapiro–Wilk normality test. The post hoc Duncan test was then performed with a 95% confidence interval (CI), following the suggestion from previous reports [13]. All analyses were carried out using the SPSS software (SPSS Inc., Chicago, IL, USA).

## 3. Results and Discussion

### 3.1. Platelets

The K0 group, the negative control, was found to possess the highest platelet value (Table 2). The pathogenic activity of *S. pyogenes* reduced the number of platelet cells in the K1 group after infection on the first day. The reduced platelet count was associated with the aggregation of platelets in the infection sites following platelet binding by haemostasis-related receptors [14]. As for the K2 and K3 groups receiving yogurt therapy, the number of platelets was higher in the K2 group as compared with that in the K1 group. It suggests the ability of yogurt containing probiotics in maintaining the number of platelets. Probiotics could attach to epithelial cells and release several free amino acids and synthesize vitamins needed by the growth of the host platelets [15]. It was corroborated by the observation on day 7 and 14, where the K2 and K3 groups had a relatively higher number of platelets. The K2 group (14 days therapy) was found to have a higher platelet count than the K3 (7 days therapy), indicating that longer probiotic therapy may induce clinical benefits. Interestingly, the number of platelets keeps increasing 7 days post probiotic therapy (K3 group) indicating the longer effect of the probiotic in maintaining the platelets (Table 2) [11].

### 3.2. Leukocyte

The number of leukocytes was found to be lower in the infected group (K1) than in groups receiving no treatment (K0) (Table 2). This observation is similar to the findings from the previously reported study [11]. In a normal rat, the range of leukocyte count is 2000–10000 cells/*μ*L [16]. Although the number of leukocytes in the K1 group remained within normal limits, the leukocyte count was statistically lower (*p* < 0.05) as compared with other rat groups. The K2 Group had leukocyte counts higher or similar to that of the K0 group, indicating the effectiveness of 14-days probiotic therapy. It could be attributed to the role of probiotics as immunomodulators, hence improving the leukocyte count [17, 18]. Several strains of lactic acid bacteria that are probiotics stimulate the immune system, e.g., repairing macrophages, increasing antibodies, and controlling infection [19–22]. Nonetheless, the administration of the probiotic did not significantly affect the number of leukocytes in the K1 group, suggesting that a treatment duration of 7 days was insufficient (Table 2).

### 3.3. Lymphocyte

The normal value of mouse lymphocytes is 60–75% [16, 23]. The K0 group was a negative control with lymphocyte count (%) on days 1, 7, and 14, namely 32.50 ± 0.71, 30.00 ± 0.00, and 31.00 ± 0.00, respectively (Table 2). The group of infected rats without yogurt (K1) on days 1, 7, and 14 had lymphocyte counts (%) of 40.00 ± 1.41, 45.50 ± 0.71, and 43.00 ± 1.41, respectively. This shows that the K1 group has a significantly higher number of lymphocytes than the K0 group (*p* < 0.05). It suggests the role *S. pyogenes* infection in elevating the number of lymphocyte cells, as suggested previously [11]. The number of lymphocytes increased to 45.50 ± 0.71% in the first week. It explains that lymphocytes play a role in forming antibodies to protect the body from infection [24]. In the K2 and K3 groups, yogurt administration for seven days until the 14^th^ day decreased the number of lymphocytes. Only in the K3 group the number of lymphocytes slightly increased to 34.50 ± 2.12% on the 7^th^ day, although it was not significantly different from the negative control group (Table 2). These results indicate that the content of probiotics in yogurt increases the immune system, improving lymphocyte profiles, which are in line with previous findings [25, 26].

### 3.4. Neutrophil

The K0 group was a negative control with the number of neutrophils (%) on days 1, 7, and 14 reaching 25.50 ± 0.71, 24.50 ± 0.71, and 24.00 ± 1.41, respectively (Table 2). Meanwhile, the K1 group experienced an elevated number of neutrophils following the course of the infection. The number of neutrophils in the K1 group was the highest among all groups (*p* < 0.05). The role of *S. pyogenes* in elevating the neutrophil count had been witnessed in a previous study [11]. The administration of probiotics in this present study was proven to be capable of restoring the neutrophil counts to the normal range (Table 2). Restoring the number of neutrophils is important since it has a role in preventing bacterial infection in the body [27].

### 3.5. Monocyte

The K0 group was a negative control with monocyte a count (%) on days 1, 7, and 14 reaching 2.70 ± 0.42, 2.55 ± 0.64, and 2.60 ± 0.56, respectively (Table 2). *S. pyogenes* infection in the K1 group caused a significant increase in the monocyte count, observed on day 7 and 14 (6.15 ± 1.20 and 6.45 ± 0.63%). The normal range of the monocyte count itself is 1–6% [16]. The exacerbated monocyte count following the *S. pyogenes* infection is in agreement with the findings from a previous study [11]. Monocytes have a phagocytic function. Monocytes are specific initial defense cells in rats that get rid of foreign objects that enter the body [28]. Meanwhile, the monocyte counts in K2 and K3 groups in all observations were found to be insignificant as compared with the negative control (K0) with *p* > 0.05 (Table 2).

## 4. Conclusions

The efficacy of probiotic therapy against *S. pyogenes* infection in rats has been proven by the recovery of haematological profiles. Probiotic treatment as short as 7 days could result in improving platelet and leukocyte counts 7 days posttreatment. Longer treatment duration was proven to contribute to the efficacy of probiotics in inhibiting the pathogenic activity of *S. pyogenes*. Clinical studies were warranted on the efficacy of probiotic therapy for *S. pyogenes*-induced throat inflammation.

## Figures and Tables

**Table 1:** Treatment group on experimental rats [11].

No.	Rat groups	Treatment
1	Negative control (K0)	Normal rats were only fed and aquadest. A blood test was conducted on the 1^st^, 7^th^, and 14^th^ days.

2	Bacterial infection (K1)	Rats were fed and infected with *S. pyogenes* bacteria in the throat on the first day. A blood test was conducted on the 1^st^, 7^th^, and 14^th^ days.

3	Administering yogurt and bacterial infection (K2)	Rats were fed and infected with *S. pyogenes* bacteria in the throat on the first day. They were also administered with yogurt for 14 days at a dose of ±1.00 mL. A blood test was conducted on the 1^st^, 7^th^, and 14^th^ days.

4	Administering yogurt and bacterial infection (K3)	Rats were fed and administered with yogurt at a dose of ±1.00 mL for seven days, then, *S. pyogenes* bacteria were infected in the throat. A blood test was conducted on the 1^st^, 7^th^, and 14^th^ days.

**Table 2:** Results of rat's blood haematology analysis.

Parameter	Day	Treatment groups			
		K0	K1	K2	K3
Platelets (cells/*μ*L)	1 7 14	355500 ± 7778.2^b^ 362500 ± 24748.7^b^ 324500 ± 31819.8^b^	96000 ± 1414.2^a^ 92500 ± 3535.5^a^ 91500 ± 2121.3^a^	247500 ± 22627.4^c^ 390000 ± 12727.9^b^ 330000 ± 4242.6^b^	238000 ± 99649.4^c^ 230000 ± 70710.6^c^ 324500 ± 7778.2^b^

Leukocyte (cells/*μ*L)	1 7 14	10750 ± 353.5^b^ 10800 ± 424.2^b^ 10850 ± 353.5^b^	8200 ± 141.4^a^ 8250 ± 353.5^a^ 8000 ± 1272.8^a^	10700 ± 989.9^b^ 10550 ± 494.9^b^ 10500 ± 565.6^b^	9500 ± 565.6^a,b^ 9550 ± 353.5^b^ 9800 ± 565.6^a,b^

Lymphocyte (%)	1 7 14	32.50 ± 0.71^a^ 30.00 ± 0.00^a^ 31.00 ± 0.00^a^	40.00 ± 1.41^b^ 45.50 ± 0.71^c^ 43.00 ± 1.41^b^	32.00 ± 1.41^a^ 29.50 ± 0.71^a^ 30.00 ± 1.41^a^	30.50 ± 0.71^a^ 34.50 ± 2.12^b,a^ 29.00 ± 2.82^a^

Neutrophil (%)	1 7 14	25.50 ± 0.71^a^ 24.50 ± 0.71^a^ 24.00 ± 1.41^a^	35.50 ± 0.71^c^ 39.00 ± 1.41^c^ 37.50 ± 2.12^c^	29.50 ± 2.12^b^ 25.00 ± 1.41^a^ 23.00 ± 1.41^a^	27.50 ± 0.71^a^ 33.50 ± 0.71^b^ 29.00 ± 1.41^b^

Monocyte (%)	1 7 14	2.70 ± 0.42^a^ 2.55 ± 0.64^a^ 2.60 ± 0.56^a^	5.55 ± 0.78^a^ 6.15 ± 1.20^b^ 6.45 ± 0.63^b^	2.80 ± 0.28^a^ 2.55 ± 0.78^a^ 2.95 ± 1.34^a^	3.30 ± 0.98^a^ 4.50 ± 0.71^a^ 4.00 ± 0.00^a^

^a, b^Similar letter notation indicates no significant difference in Duncan's test with a value of 5%.

## Data Availability

The data used to support the findings of this study are available from the corresponding author upon request.
